# Microarray characterization of gene expression changes in blood during acute ethanol exposure

**DOI:** 10.1186/1755-8794-6-26

**Published:** 2013-07-25

**Authors:** Doris M Kupfer, Vicky L White, David L Strayer, Dennis J Crouch, Dennis Burian

**Affiliations:** 1 Civil Aerospace Medical Institute, AAM 610, Federal Aviation Administration, Bioaeronautical Sciences Research Laboratory, Oklahoma City, OK 73169, USA; 2 Department of Psychology 380 S. 1530 E. BEHS 502, Salt Lake City, UT 84112, USA; 3 Utah Toxicology-Expert Services, Sandy, UT 84092, USA

**Keywords:** Ethanol, Blood, Gene expression, Biomarkers, Microarray

## Abstract

**Background:**

As part of the civil aviation safety program to define the adverse effects of ethanol on flying performance, we performed a DNA microarray analysis of human whole blood samples from a five-time point study of subjects administered ethanol orally, followed by breathalyzer analysis, to monitor blood alcohol concentration (BAC) to discover significant gene expression changes in response to the ethanol exposure.

**Methods:**

Subjects were administered either orange juice or orange juice with ethanol. Blood samples were taken based on BAC and total RNA was isolated from PaxGene™ blood tubes. The amplified cDNA was used in microarray and quantitative real-time polymerase chain reaction (RT-qPCR) analyses to evaluate differential gene expression. Microarray data was analyzed in a pipeline fashion to summarize and normalize and the results evaluated for relative expression across time points with multiple methods. Candidate genes showing distinctive expression patterns in response to ethanol were clustered by pattern and further analyzed for related function, pathway membership and common transcription factor binding within and across clusters. RT-qPCR was used with representative genes to confirm relative transcript levels across time to those detected in microarrays.

**Results:**

Microarray analysis of samples representing 0%, 0.04%, 0.08%, return to 0.04%, and 0.02% wt/vol BAC showed that changes in gene expression could be detected across the time course. The expression changes were verified by qRT-PCR.

The candidate genes of interest (GOI) identified from the microarray analysis and clustered by expression pattern across the five BAC points showed seven coordinately expressed groups. Analysis showed function-based networks, shared transcription factor binding sites and signaling pathways for members of the clusters. These include hematological functions, innate immunity and inflammation functions, metabolic functions expected of ethanol metabolism, and pancreatic and hepatic function. Five of the seven clusters showed links to the p38 MAPK pathway.

**Conclusions:**

The results of this study provide a first look at changing gene expression patterns in human blood during an acute rise in blood ethanol concentration and its depletion because of metabolism and excretion, and demonstrate that it is possible to detect changes in gene expression using total RNA isolated from whole blood. The analysis approach for this study serves as a workflow to investigate the biology linked to expression changes across a time course and from these changes, to identify target genes that could serve as biomarkers linked to pilot performance.

## Background

As part of civil aviation safety research into aeromedical factors impacting flight safety, we have undertaken a time course study on the effects of acute ethanol exposure to the legal intoxication level of blood alcohol concentration (BAC), 0.08 g/dL (0.08% wt/vol), on human gene expression in non-chronic users. This is a novel study to identify gene expression patterns in whole blood messenger RNA and discover biomarkers linked to BAC.

Mandatory ethanol testing was implemented for U.S. commercial pilots in 1995. For the period 1995–2000 alcohol violations, occurrences of an alcohol level exceeding the Federal Aviation Administration (FAA) limit of ≥0.04%, were attributed to 0.13% of fatal aviation accidents [1]. This number suggests that alcohol misuse is a relatively rare occurrence in the commercial aviation setting. To date, alcohol has not been implicated as the probable cause in any fatal crash involving a U.S. major airline; however, there are instances of commercial pilots reporting for duty impaired with BAC ≥0.04% [2,3].

The current FAA regulations prohibit anyone from acting as a crewmember within eight hours of consuming alcohol or while having a BAC ≥0.04%. However, a number of reports addressing the carry-over effect of alcohol have shown a performance loss to remain after the BAC has returned to less than 0.02% [4]. Communication performance decrement was still found eight hours post 0.08% BAC, and an ethanol effect on the vestibular and visual systems has been shown to persist for up to several days after BAC returned to zero (reviewed in [5]). Additionally, two studies [4,6] found that testing at eight hours post 0.1 or 0.08% BAC showed variability in performance suggesting a range of susceptibility to ethanol. The authors suggested that an arbitrary eight hour flying prohibition does not take into account the amount consumed or individual differences in metabolism and recovery.

General (private) aviation accounts for more than 90% of all aviation accidents [7]. For the period 1994–2000, 11.5% of alcohol-related general aviation crashes were linked to a pilot with a DWI (driving while intoxicated) history [8]. No routine alcohol testing is required for general aviation pilots. The level of fatal general aviation crashes attributed to alcohol impairment has decreased from 30% of pilots with a BAC ≥0.04% in the early 1960s to 8% in the 1990s [9]. However, a study of alcohol-related fatal crashes between 1985 and 2000 in Maryland, New Mexico, and North Carolina showed that 11% had positive BACs and 6% had BACs exceeding 0.04% [10]. The FAA Toxicology and the Document Information Workflow System databases contain records of the 2391 certified pilots involved in fatal accidents for the period 2000–2007. These records show that 215 pilots tested positive post-mortem for alcohol and had drug or alcohol offenses. Twenty-three of the 215 were confirmed to have consumed ethanol prior to the fatal incident. In eleven of these cases, the National Transportation Safety Board linked the accident cause to alcohol impairment [11]. These studies suggest that in general aviation, flight impairment due to alcohol consumption is still a safety concern.

FAA forensic toxicological testing detected ethanol above the legal cutoff of 0.04% in 7% of fatal general aviation accidents during 2000–2007 [11]. To differentiate antemortem from postmortem alcohol in positive cases, the FAA (as of November, 2012) uses either the standard practice of tissue distribution ratios of alcohol present in blood, vitreous humor, and tissues or, if urine is available, the ratio of the serotonin metabolites, 5-HTOL and 5-HIAA [12,13]. Unfortunately, in approximately 30% of ethanol-positive cases, distribution ratios are inconclusive and FAA toxicologists are unable to differentiate between ingested and other sources of the detected ethanol (R. Lewis, personal communication, November 14, 2012).

Ethanol ingestion impacts human metabolism, lowering blood glucose by stimulating the glucose-stimulated insulin early secretion response, which can result in a transient hypoglycemia [14]. Metabolism of ethanol occurs primarily in the liver and results in production of intermediate acetaldehyde and then acetate, which can be utilized by other tissues. This oxidation results in a decrease in the NAD+/NADH ratio (reviewed in [15]). Ethanol exposure increases the level of reactive oxygen species (ROS) in part because of the shift in NAD+/NADH ratio and the action of cytochromes p450 and CYP2E1 [16].

The impact of acute ethanol consumption on the innate immune system and inflammation is well-documented and includes suppression of proinflammatory cell activation in a dose-dependent manner [17]. Nuclear factor κB (NF-κB) is a central mediator of the innate immune response [18] whose activity is reduced in the presence of ethanol, in turn affecting levels of pro-inflammatory cytokines, TNF and IL-1B at the transcription level [19,20].

A major kinase signaling cascade is anchored by the p38 mitogen activated protein kinase (MAPK). It is activated by a variety of stressors and inflammatory cytokines [21]. p38 MAPK regulates a diverse set of downstream transcription factors, pathways, and cell functions including cytokine production, cell proliferation, differentiation, and apoptosis including transcription activity of NF-κB. Acute ethanol exposure has a negative affect on p38 MAPK activity, leading to decreased NF-κB transcriptional activity, lower levels of TNF and of proinflammatory cytokine production [22,23], and, ultimately, neutrophil and granulocyte migration to sites of inflammation [17]. Conversely, ethanol induces oxidative stress by increasing levels of ROS ([16] that can enhance p38 MAPK activity. p38 MAPK phosphorylates p300, which in turn acetylates the RelA component, increasing NF-κB transcription activity [23]. Cytochrome C and calcium are inter-organellar messengers of apoptosis (reviewed in [24]). The p38 MAPK pathway is linked to apoptosis through ROS, mediating mitochondrial dysfunction, triggering the release of cytochrome C, followed by calcium release from the ER [25].

Transcription factor STAT3 phosphorylation increases in the presence of acute ethanol because of increased activity of Src kinases [26]. IL-10, an anti-inflammatory cytokine, is a target of the src-STAT3 pathway and shows an increase in transcription in the presence of acute ethanol [19]. STAT3 also is an activator of Suppressor of Cytokine Signaling 3 (SOCS3), and SOCS1, negative regulators of cytokine signaling [27]. Additionally, acute ethanol exposure has been shown to have a reproducible and negative affect on the ability of both monocytes and dendritic cells to stimulate T-cell antigen presentation function [28].

Our approach for this study was to evaluate changes in gene expression levels, using RNA extracted from whole blood, across a five-point time course as ethanol entered the blood system, reached a level of 0.08 g/dL (0.08% BAC), and returned to 0.02% BAC, the lowest concentration of breathalyzer detection. By microarray analysis, we examined gene expression changes and evaluated the resulting genes of interest (GOIs) for ethanol-related biological relevance, identifying sets of genes to serve as potential biomarkers for alcohol-related effects.

## Methods

### Subject profiles

Nine age-matched subjects for the ethanol study were recruited by D. L. Strayer, Department of Psychology, University of Utah, Salt Lake City UT. Institutional Review Board approval to perform research on human subjects was gained from boards at both the University of Utah and the FAA Civil Aerospace Medical Institute (CAMI). The study was conducted at the Department of Psychology, Salt Lake City, Utah. Informed consent was obtained by investigators at the Department of Psychology. The control experiment to establish the effects of drinking orange juice only was conducted at the CAMI. Five age-matched male subjects were recruited at the University of Central Oklahoma. Institutional Review Board approval was granted from boards at both the University of Central Oklahoma and the CAMI. Informed consent was obtained by investigators at the CAMI for the five subjects.

### Sample collection and preparation

Subjects drank 125 mL of an orange juice and 80 proof vodka mixture calculated to achieve a blood alcohol concentration of 0.08% wt/vol [29]. Blood Alcohol Concentrations (BACs) were verified using infrared spectrometry breath analysis (Intoxilyzer 5000, CMI Inc; Ownsboro, KY, [30]). Blood samples were collected into PAXgene™ Blood RNA tubes (Cat. # 762165, Qiagen US; Valencia, CA) at five time points corresponding to BAC, baseline = BAC1, 0.04% = BAC2, 0.08% = BAC3, 0.04% = BAC4 during recovery, and 0.02% = BAC5, this last point being the lower limit of quantitation by the Intoxilyzer. Control experiment samples were collected from subjects at time points corresponding to the average collection time for the alcohol group: T1 was taken prior to drinking 125 mL of orange juice (OJ), T2 at 90 minutes; T3 at 2 hours, 49 minutes; T4 at 5 hours, 8 minutes; and T5 at 7 hours, 8 minutes.

For the alcohol group, two blood samples were collected at each timepoint. Total RNA was purified using the PAXgene™ RNA purification system with the optional on-column DNase treatment (Cat # 762164; Qiagen; Hilden, Germany) [31], and stored at −80°C. A modification in the manufacturer’s published protocol pooled the two samples from each subject timepoint at the column binding step such that total RNA was purified from one PAXgene™ column. A single blood sample was obtained from each control group subject for each timepoint in a PAXgene™ Blood RNA tube and purified according to the manufacturer’s published protocol with the on-column DNase step.

RNA quality was assessed on an Agilent Bioanalyzer 2100 (Agilent; Santa Clara, CA) using the Agilent RNA 6000 Nano Series II kit following manufacturer’s directions with 1 μL of sample and the 2100 Expert software (ver. B.02.03.SI307). Yield and A260/280 was determined on a Nanodrop 1000 spectrophotometer (Thermo Scientific; Waltham, MA) (Additional file 1).

Samples from six male subjects (S8, S13, S15, S17, S18, and S19) were used in the ethanol microarray study and samples from one female (S1) and 5 male subjects (S5, S10, S13, S17, S19) were used for qRT-PCR validation of microarray results.

Microarray target material was made using the One-cycle IVT kit (P/N 900431; Affymetrix Inc.; Santa Clara, CA) and the resulting amplified cRNA hybridized to Affymetrix HGU133plus2.0 GeneChips® according to the manufacturer’s instructions. One sample, S18-BAC4 was excluded from further analysis due to a low percent present call with MAS5.0 (Affymetrix Inc.; Santa Clara, CA) and insufficient RNA to repeat the amplification/hybridization leaving 29 samples and arrays.

Total RNA from the five orange juice (OJ) Control subjects was used to derive target material using the Ovation 3’ kit (P/N 2200, NuGen Technologies; San Carlos, CA) according to the manufacturer’s protocol and hybridized to Affymetrix HGU133plus2.0 GeneChips®.

### Data handling and analysis

To assess quality and variability in the microarray data, CEL files underwent standard QC analysis using the Affymetrix™ GeneChip Operating Software, GCOS (Affymetrix Inc.; Santa Clara, CA), including a percent present determination using MAS5.0 (Affymetrix Inc.; Santa Clara, CA) as noted in the section above. For the ethanol subjects, CEL files were imported into S + ArrayAnalyzer™, version 2.1.1 (Tibco Software Inc.; Palo Alto CA), summarized and quantile normalized using both RMA [32] and GCRMA [33] algorithms. RMA summarized data was filtered for log_2_ (RMA expression) >6 in at least six arrays; GCRMA, summarized data were filtered for log_2_ (GCRMA expression) >5 in at least six arrays. The Local Pooled Error T-test, LPE, [34] was used to test for significance of differential gene expression across all possible pairwise comparisons in both sets of summarized data. Probe sets with p < 0.05 after False Discovery Rate correction by the method of Benjamini and Hochberg [35] were further filtered for a fold change greater than 1.25 in at least one pairwise comparison.

RMA summarized data were used for analysis using Extraction and Analysis of Differential Gene Expression, EDGE [36] and the timecourse package [37] in Bioconductor [38]. The EDGE output was filtered for probe sets with q < 0.02, the EDGE-specified cutoff. From the timecourse analysis, the ranked T^2^ test scores were visualized as a histogram. Based on a leveling off of the histogram as scores reached 50, probe sets with ≥50 were considered the Timecourse50 list. This list was added to the lists derived from LPE testing and EDGE for further analysis. Duplicate probe sets and those with the Affymetrix x*_*at designation were removed and where a gene was represented by multiple probe sets, the probe set with the greatest fold change was retained.

CEL files from OJ Control samples were analyzed using the same three methods: LPE, EDGE and timecourse. Since the control subjects were not the same as the ethanol subjects, to generate a comprehensive list of genes and functional pathways responding to orange juice alone thereby minimizing false positives in the alcohol-responsive gene list, a significance score of T^2^ ≥ 25 was applied to the timecourse analyzed control data. Probe sets found on both the ethanol and OJ lists were removed from the alcohol list.

qRT-PCR validation of genes across a range of fold-changes was used to refine endpoint settings for all three analysis methods (Results and see below). Cutoff limits were set at 1.53 fold-change for LPE derived genes, q ≤ 0.0017 EDGE derived genes and T^2^ > 82.83 for timecourse derived genes resulting in a list of 203 probe sets for further analysis. To cluster probe sets by temporal expression pattern, Z-transformed expression values for the probe sets were used as input for K-means fuzzy-clustering [39].

**Figure 1:**
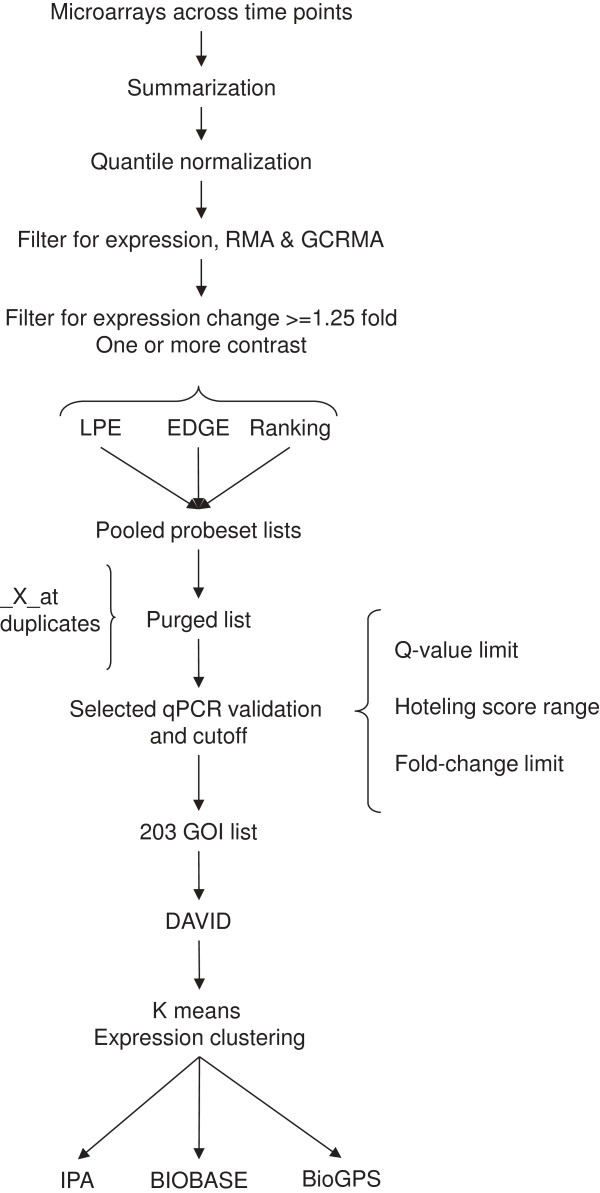
**Microarray data analysis pipeline.** Analysis pipeline for control and ethanol exposed microarray data sets.

The entire probe set list and the individual expression pattern-clustered probe sets, were analyzed with Ingenuity Pathway Analysis, IPA (version 8.7, Ingenuity® Systems, Inc., http://www.ingenuity.com; Redwood City, CA) and the Database for Annotation, Visualization, and Integrated Discovery, DAVID [40]. The BioGPS database was used to evaluate tissue-specific gene expression using the Human U133A/GNF1H Gene Atlas dataset, (GEO-GSE1133, [41,42] ). The BIOBASE [43] ExPlain™ [44] Mammalian Module 3.0 was used with the application Match™ to examine the promoter regions of the cluster genes for transcription factor binding matrices using the BIOBASE TRANSFAC® database. The RMA summarized data set filtered for average log_2_ (expression) > 6 and with the 203 candidate genes removed was used for the No-set (background). The vertebrate_h0.01 profile was used. High-specific matrices with cut-offs minFP were used for a 1200 base promoter window from −1000 to 200. Both cut-off and window position were optimized with a p-value threshold of 0.001. The Match matrix output was filtered for a Yes/No ratio of >1.5, P-value <0.01 and Matched promoters P-value <0.01. The weight matrices profile was used to create a transcription factor gene set and filtered for human specific factors. The gene set was mapped on canonical pathways using the BIOBASE Transpath application using P-value <0.01, minimal hits to group of two. A Transpath gene set linking the transcription factors to pathways was generated for each cluster. The resulting output was examined for signaling pathways and transcription factors predicted to affect genes on our list. Figure 1 shows the analysis pipeline.

The microarray data is publicly accessible and has been assigned series number GSE20489 in the Gene Expression Omnibus (GEO) [45] and can be freely accessed from their website.

### Quantitative PCR

Fifty nanograms of total RNA from each of six ethanol subjects, S1, S5, S10, S13, S17, and S19, were reversed transcribed and amplified for use with qRT-PCR with the Ovation 3’ amplification kit (P/N 2200, NuGen Technologies; San Carlos, CA) according to the manufacturer’s protocol. Candidate GOIs for qRT-PCR were chosen from the EDGE-generated list (IMPA2, TMEM8 and CDA) and from the pooled LPE list (ANKRD28, EVI2A, FKBP5, LR8). The final GOI, RNF11, was on both lists.

Candidate normalizer genes were filtered from RMA-summarized, non-normalized microarray expression data with coefficient of variance < 0.2 and average log2 expression >5.5 across all 29 chips and further refined with the analysis tools NormFinder [46] and geNorm [47]. geNorm pairwise variation showed that three normalizers were sufficient for the study. The three genes selected, ITGA5, SCAMP2, and PLCG2, had NormFinder stability rankings in the top 20% of the candidates, 0.045, 0.045 and 0.048, respectively, and are functionally non-redundant. Genes with better stability rankings were not good candidates for primer design.

Primers for both normalizer genes and GOIs were designed for detection by SYBR-Green using Beacon Designer 7.0 (PremierBiosoft; Palo Alto, CA) (Additional file 2) and obtained from Integrated DNA Technologies (Coralville, IA). Primer pairs were rigorously screened for qRT-PCR reaction efficiency greater than or equal to 93% and the presence of a single product by electrophoresis on a Bioanalyzer DNA 1000 chip (Agilent; Santa Clara, CA). qRT-PCR was performed on a MX3005P cycler (Stratagene; La Jolla, CA) using Platinum SYBR-Green qRT-PCR SuperMix UDG (P/N 11733046, Invitrogen; Carlsbad, CA) in 25 μL reactions according to the enzyme manufacturer’s recommendations and optimal primer annealing temperature for each primer pair. A noRT experiment with TMEM8 revealed all samples had 10 Cts or greater difference compared to the experimental samples. Differential expression analysis of qRT-PCR data was performed with REST2009 [48] after normalization to the geometric mean of normalizer expression values.

## Results and discussion

### Microarray data analysis and candidate gene list derivation

We report microarray derived gene expression changes over a BAC profile to a maximum of 0.08% after ingestion of an alcohol cocktail of orange juice (OJ) and vodka. A control group was administered only orange juice to distinguish genes and biological pathways responding to the OJ from those responding to the alcohol. Five samples were taken from each subject (BAC1-5), a baseline sample and then at BAC levels, or for the control group, the time matched equivalent, of 0.04%, 0.08%, and recovery samples at 0.04% and 0.02%, the lowest concentration detectable by breathalyzer (Methods).

Because the experimental conditions were performed with different groups of subjects, we used a conservative analysis model that included three microarray data analysis tools - LPE t-test [34], EDGE [36] and timecourse [37] (Methods) - to identify candidate genes. The LPE t-test was applied across all 10 possible pairwise timepoint comparisons and the lists of significant probe sets pooled. From the ethanol data, the LPE list contained 171 probe sets, the EDGE list 63 probe sets, and the Timecourse50 list 452 probe sets.

When applied to the OJ data, the LPE t-test found 22 differentially expressed probe sets, one of which was in common with the ethanol list. The EDGE analysis found no significantly changing probe sets. In the timecourse analysis, 23 probe sets above the test score cutoff of 25 were found in common with the ethanol list. These 24 probe sets were removed from the ethanol list. The 19 genes represented by these probe sets were examined in DAVID and IPA to determine pathways responding to OJ. GO_FAT results from DAVID showed that eight genes are involved in translation and translation elongation. Therefore, the “protein translation” biological process was not considered in further analysis of the ethanol data.

#### Quantitative PCR validation and establishment of cut-off values

qRT-PCR was used to validate the microarray results for direction and fold-change across the experiment. Four genes were selected from the LPE list: ANKRD28, EVI2A, FKBP5, LR8, and the EDGE list: IMPA2, TMEM8, CDA and RNF11 (Table 1). Only ANKRD28 and LR8 were not on the Timecourse50 list. ITGA5, SCAMP2 and PLCG2 were selected as normalizers based on microarray expression data across all 29 chips (Methods).

**Table 1:** Genes used in the qPCR validation study

**Probeset**	**Gene**	**EDGE**		**LPE**			**Timecourse**
		**Q-value**	**Q-rank**	**P-value (= < 0.05)**	**Microarray fold change**	**Blood alcohol concentration (BAC) significant point comparisons**	**T**^**2 **^**hoteling score**
**Normalizers**							
201389_at	ITGA5	0.0063	312	NA^	NA	NA	**12.12**
204613_at	PLCG2	0.0648	2817	NA	NA	NA	**21**
218143_s_at	SCAMP2	0.0161	829	NA	NA	NA	**8.37**
**LPE selected genes**							
226025_at	ANKRD28	0.0114	563	0	1.689	BAC 2 vs 5	**25.97**
204774_at		0.0452	2135	0.021	1.45	BAC 2 vs 5	95.59
				0	1.64	BAC 3 vs 4	
				0	1.95	BAC 3 vs 5	
224840_at	FKBP5	0.0347	1715	0.009	−1.54	BAC 1 vs 4	326.231
				0.044	−1.53	BAC 1 vs 5	
				0.025	−1.57	BAC 2 vs 4	
220532_s_at	LR8	0.287	9699	0	−1.25	BAC 1 vs 4	**9.52**
**EDGE selected genes**							
203126_at	IMPA2	0.0013	3	NA	NA	NA	82.828
221882_s_at	TMEM8	0.0013	6	NA	NA	NA	85.406
205627_at	CDA	0.0013	11	NA	NA	NA	132.76
208924_at	RNF11^*^	0.0017	51	0.006	1.76	BAC2 vs 5	208.403

*RNF11 is found on all three lists.

^*NA* Not Applicable, P-value > 0.05, not on gene list for given analysis method.

Bold-Not on the TIMECOURSE50 list.

Comparisons across all 10 possible pair-wise BAC contrasts were performed using Relative Expression Software Tool, REST ([48], Methods). The direction of change was found to be the same for the microarray and qRT-PCR results with the single exception of the BAC 4 versus 5 contrast for ANKRD28, where the microarray results showed an increase rather than the decrease seen in the qRT-PCR comparison (Table 2); however, this was not a statistically significant contrast in the LPE t-test (Table 1).

**Table 2:** qPCR relative expression ratios between BAC points

**Timepoint comparison**										
**GOI**	**BAC1-2**	**BAC1-3**	**BAC1-4**	**BAC1-5**	**BAC2-3**	**BAC2-4**	**BAC2-5**	**BAC3-4**	**BAC3-5**	**BAC4-5**
**EVI2A**		−1.7			−2			1.76	1.63	
**RNF11**	−1.45				1.981	1.87	1.808	1.35		
**ANKRD28**			1.477			2.282	1.671	1.72		**−1.435**
**FKPB5**			−1.65	−1.439		−1.5		−1.58		
**TMEM8**				−1.4			−1.432			
**CDA**				−1.42			−1.67		−1.55	−1.35
**IMPA2**	−1.28		−1.34	−1.3				−1.309		
**LR8**										

*GOI*, Gene of interest.

*BAC*, blood alcohol concentration, refers to the level of alcohol present in subject at time of blood sampling, see M&M.

A negative value means a decrease in relative expression for the second timepoint relative to the first.

Bold-direction of change disagrees with microarray analysis.

With two exceptions, all other statistically significant contrasts from microarray data were validated by qRT-PCR. LR8, significant in a single contrast at a fold-change level of 1.25 (Table 1), could not be confirmed, and EVI2A could not be confirmed at one of its three significant contrasts (BAC2 vs. 5, Table 1). Therefore, for further characterization of the biological response to ethanol, the limit of detection for genes coming from the LPE analysis was set at a fold change of 1.53, based on confirmation of FKBP5 (Tables 1 and 2). Likewise, based on the observation that the largest q-value from the EDGE analysis that could be validated by qRT-PCR was 0.0017, the gene list was filtered to remove EDGE-list genes above that level. Similarly, the lowest validated timecourse score was 82.83, based on IMPA2 (Table 1). Genes below this level were removed. The resulting merged list from the three analyses contained 203 GOIs.

#### Functional analysis of the genes of interest (GOIs)

To obtain an overall picture of pathways that respond to ethanol ingestion, the complete list of 203 GOIs was assessed using IPA. Functional categories included immune and inflammatory response, hematological system development and function, hepatic system disease, carbohydrate metabolism, cell death, cell-cell signaling, and nucleic acid and amino acid metabolism. Using DAVID, the GOIs clustered into functional categories including immune system development, protein catabolism, and S100A EF hand proteins. An examination for over-represented GO terms from DAVID in the candidate gene list identified 21 genes with immune response, 16 with defense response and eight with innate immune response.

To further examine the temporal response to ethanol exposure, we clustered the GOIs by expression pattern. Seven clusters were formed by 199 of the genes; four were unclustered. The seven clusters were analyzed using IPA, BIOBASE, and BioGPS (Figure 1 and Methods).

#### Cluster 1

**Figure 2:**
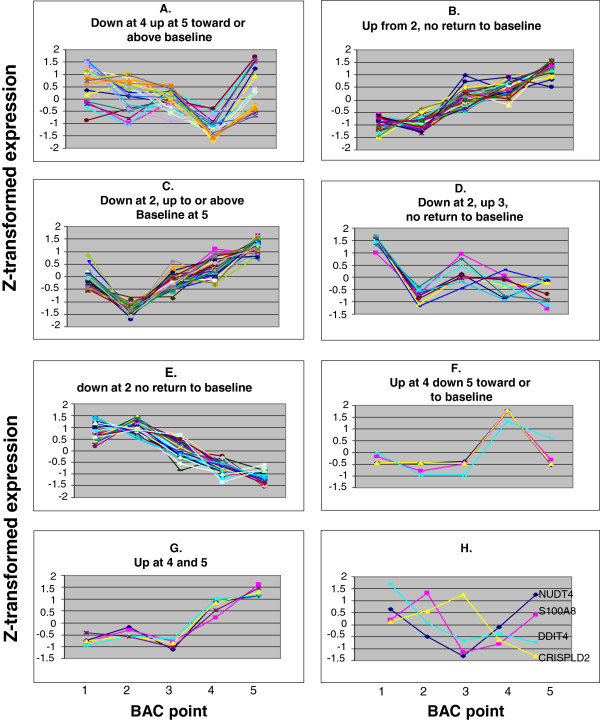
**Expression pattern clustering.** Each panel **A-H** shows the expression pattern for individual members of each gene cluster based on microarray expression data for the gene probe sets at each of the five BAC points. **A**. Cluster 1, 23 members with expression pattern down at 4 up at 5 toward or above baseline. **B**. Cluster 2, 44 members with expression pattern up from 2, no return to baseline. **C**. Cluster 3, 47 members with expression pattern down at 2, up to or above baseline at 5. **D**. Cluster 4, nine members with expression pattern down at 2, up 3, no return to baseline. **E**. Cluster 5, 67 members with expression pattern down at 2 no return to baseline. **F**. Cluster 6, four members with expression pattern up at 4 down 5 toward or to baseline. **G**. Cluster 7, five members with expression pattern up at 4 and 5. **H**. Unclustered, four genes which did not fall into any cluster. Percent blood ethanol concentrations, BAC1 = 0%, BAC2 = 0.04%, BAC3 = 0.08%, BAC4 = 0.04%, BAC5 = 0.02%.

Genes in this cluster exhibit a decreased expression level at BAC4 followed by a sharp increase at BAC5 (Figure 2), suggesting that these genes constitute a late response of increased expression well after alcohol levels begin to decrease. Of the 23 genes in Cluster 1, IPA created a single network from 14 members with the top biological functions of Infectious Disease, Cell Signaling, and Small Molecule Biochemistry (Table 3).

**Table 3:** Ingenuity pathway analysis* results

**Input**	**Target genes**	**Target members**	**Score-log p-value**	**Network functions**
Cluster 1	23	14	37	Infectious Disease, Cell Signaling, and Small Molecule Biochemistry
Cluster 2	44	40	94	Cellular assembly and Organization, DNA Replication, Recombination and Repair, Cell Cycle, Immune Deficiency
Cluster 3	47	36	78	Cell-To-Cell Signaling and Interaction, Small Molecule Biochemistry Cell Cycle, Cellular Function and Maintenance, Hematopoiesis and Hematological System Function
Cluster 4	9	7	13	Cell Death, Small Molecule Biochemistry Infectious Disease Response, Cellular Growth and Proliferation
Cluster 5	67	39	78	Immune Cell Trafficking, Hematological system Development and Function, Apoptosis, Cell signaling, Small Molecule Biochemistry
		20	32	Cellular Growth, Proliferation and Development, Gene Expression Carbohydrate Metabolism
Cluster 6	4	2	7	Immune response
Cluster 7	5	4	11	Gene Expression

*Ingenuity Pathway Analysis Ver 14197757, Ingenuity Systems Inc.

Interactions within the network include RIOK3, a GOI that regulates NF-κB [49], which in turn interacts with the GOIs BAX, a blood exclusive, stress-induced proapoptotic factor [50], UBR5 the E3 ubiquitin ligase linked to apoptosis [51], and KLF3, a hematopoietic transcription factor important in apoptosis and the inflammatory response [52]. Transcription regulator TP53 levels are regulated by the GOIs UBE2D3, an E2 ligase [53], BAX, and the proteoglycan VCAN, important in cell adhesion [54]. Other GOIs in the network include the lysosomal marker LAMP1, important for protein trafficking [55], ACTR2, essential for cell shape [56], the cytokine regulator CNPY3 [57], PF4V1, a hematopoietic chemokine and histone methylation factor [58], and MPHOSPH8 a transcription regulator involved in DNA methylation [59].

**Figure 3:**
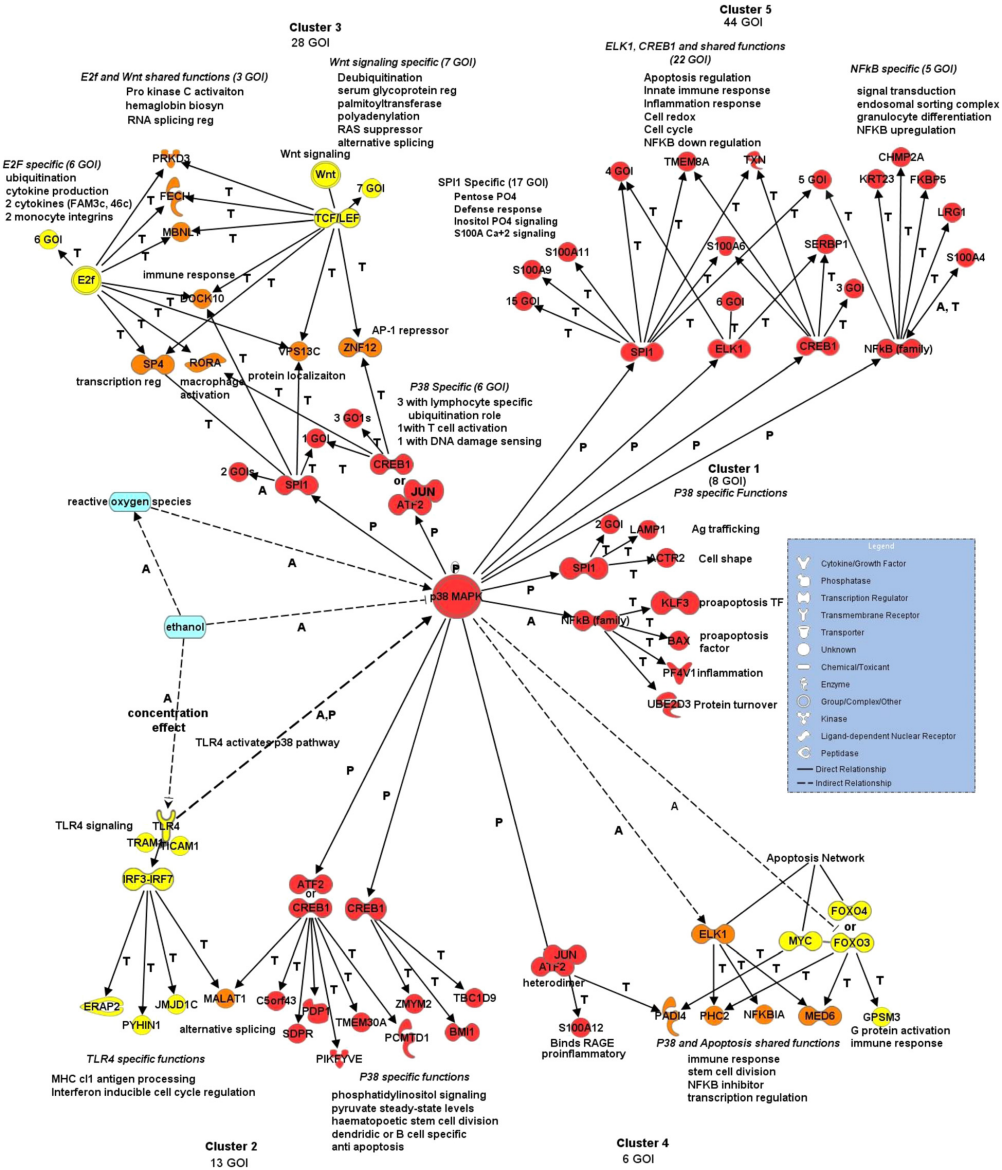
**BIOBASE-derived signaling network centered on p38 MAPK.** Summarized BIOBASE analysis results for the GOI clusters. Red circle, p38 MAPK, red molecules directly linked to the p38 MAPK pathway are BIOBASE-identified transcription factors, other red molecules GOI transcription factor targets. In yellow are non-p38 MAPK pathway transcription factors and their target GOIs. In orange are transcription factors and GOIs shared by more than one pathway. Blue indicates ethanol and ROS (reactive oxygen species). Interaction labels: A activation, P phosphorylation, T transcription. Legend: shapes define molecule types and interactions.

**Figure 4:**
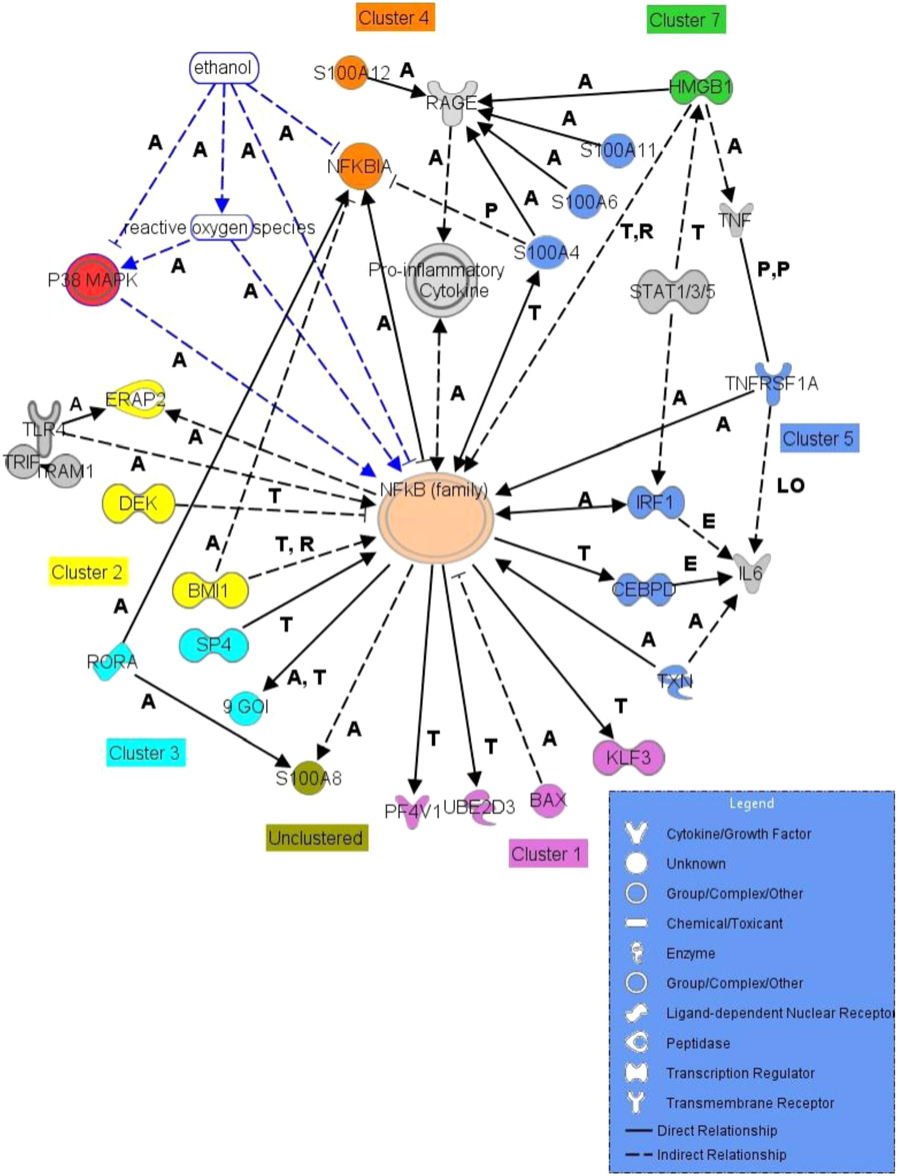
**GOI links to NF-κB determined through the analysis.** Gray non GOI factors, blue outlined Ethanol and Reactive Oxygen Species, red p38 MAPK, pink NF-κB. Other colors indicate GOI cluster membership. Purple is Cluster 1, yellow Cluster 2, turquoise Cluster 3, orange Cluster 4, blue Cluster 5, green Cluster 7. Arrows indicate activation and direction of effect, Bar indicates inhibition and direction of effect, Interaction labels: A, activation, P phosphorylation, T transcription, E expression, PP protein-protein binding, S secretion, LO localization. Legend: shapes define molecule types and interactions.

Transcription Factor Binding Site (TFBS) analysis with BIOBASE found binding sites for NF-κB in BAX, KLF3, UBE2D3, and PF4V1. LAMP1, ACTR2, and the poorly annotated genes TMEM165 and MGC2752 contain binding sites for the ETS protein SPI-1, important in lymphoid and B-cell development ([60,61]. The BIOBASE analysis reinforces the NF-κB link detected in IPA and implicates the p38 MAPK pathway and its regulation of NF-κB (Figures 3 and 4).

#### Cluster 2

Cluster 2 genes increase expression at BAC 3, 4, and 5 from BAC 1 or 2 with no return to baseline (Figure 2). IPA created a single network that includes 40 of the 44 genes in the expression cluster. Top network functions are Cellular Assembly and Organization, DNA Replication, Recombination and Repair, and Cell Cycle (Table 3). Insulin, a key sensor of glucose levels, is found in this network with connections to the GOI PDP1, an activator of pyruvate dehydrogenase [62], and PIKFYVE, a member of the inositol phosphate metabolism pathway under positive regulation by insulin [63]. Pro-inflammatory cytokine, IL-6, is a central non-GOI member of the network linked to insulin and regulating GOIs TBC1D9, a GTPase factor expressed in dendritic cells [42], BMI1, a factor in stem-cell pluripotency [64] and PARP8, involved in post-translational modification [65]. The heterotrimeric GTPase subunit GNAQ impacts the ERK and Akt signaling pathways. HNF4A, a non-GOI transcription factor regulating genes involved in glucose metabolism and homeostasis [66], has direct protein-DNA interaction with the GOIs SEC23A, an ER-Golgi trafficking molecule [67] known to be positively regulated by insulin [68], TMEM30A, also involved in protein exit from the ER [69], NEK7, a mitotic regulator [67], the TGFB1 receptor, TGFBR1 and BMI1. NF-κB, another central non-GOI in this expression cluster, interacts with or regulates the GOIs, BMI1, GNAQ, the ER-aminopeptidase, ERAP2, and TGFBR1 (Figure 4). The non-GOI cytokine TGFB1 interacts with the aforementioned receptor TGFBR1, and BMI1.

BIOBASE TFBS analysis found seven genes carrying the binding matrix for ATF2 and/or CREB1. Phosphorylation of both is regulated by p38 MAPK [70,71]. Three blood or dendritic cell-specific genes carry a matrix binding only CREB1 ([42], Figure 3). This analysis also identified four GOIs which bind interferon regulatory factors 3 and 7 (IRF3 and IRF7), members of the Toll-like receptor 4 (TLR4) pathway (Figure 3). In summary, genes in Cluster 2 are influenced by insulin and co-regulated by factors in the p38 MAPK signaling pathway.

#### Cluster 3

The expression patterns of Clusters 2 and 3 are similar; Cluster 3 exhibits an immediate-early decrease in expression from BAC1 to BAC2 not seen in Cluster 2 (Figure 2), followed by increasing expression levels through BAC5. IPA analysis of genes in Cluster 3 resulted in a network containing 36 of the 47 members (Table 3). Transcription factor HNF4A seen in the Cluster 2 network is found here linked to six GOIs, RASA1, which promotes cell migration and adhesion [72], RORA, a T-cell specific factor [73], CUL5, a cullin expressed in lymphocytes [42], DCK, expressed in whole blood and lymphocytes [42], GFM1, a mitochondrial translation elongation factor [74], and PKA, a cellular effector of cAMP [75]. The network also includes the two GOI integrins positively regulated by calcium ion and TGFB1, ITGA4, and ITGB1, components of the innate immune response (reviewed in [76]).

BIOBASE analysis associated six Cluster 3 GOIs with the p38 pathway through JUN and two transcription factors detected in Cluster 2, CREB1 and ATF2, which can heterodimerize with JUN (Table 4). These GOIs include ZNF12, a transcriptional repressor of AP-1 [77], DNAJB14, RORA, CUL5, LYRM7, and PRKACB.

**Table 4:** Pathway-specific transcription factor profile

A. p38-related TF* profile by cluster					
**Cluster**	**1**	**2**	**3**	**4**	**5**
Transcription	NF-κB				NF-κB
Factors	SPI-1		SPI-1		SPI-1
		ATF2	ATF2	ATF2	
		CREB1	CREB1		CREB1
				ELK-1	ELK-1
			JUN	JUN	
**B. Cluster-specific TF pathway profiles**					
**Cluster**	**2**	**3**	**4**	**7**	
**Pathway Membership**	**TLR4**	**Wnt, E2F**	**Apoptosis**	**STAT binding**	
Transcription	IRF3, 7				
Factors		ETF2			
		LEF1/TCF7			
			FOXO3, 4		
			ELK-1		
			MYC		
				STAT1, 3, 5A	

*TF Transcription factors predicted by BIOBASE [43] to bind cluster members as defined in the text.

Transcription factor SPI-1, an effector of p38 MAPK signaling ([78], Table 4), regulates seven Cluster 3 GOIs, RORA, DNAJB14, DOCK10, a factor induced by IL-4 in B lymphocytes [79], SP4 a transcription factor that regulates NF-κB [80], two ubiquitination factors, USP1 and RNF6, and vacuolar protein VPS13C.

Binding sites for E2F family transcription factors are found in FECH, which catalyses the last step in heme biosynthesis [81], MBNL1, linked to insulin receptor splicing [82], UBE2J1 a dendritic cell-specific [42] ubiquitin conjugating enzyme HLTF, a transcription factor regulating cytokine production [83], PRKD3 a B cell protein kinase [84], FAM46C and FAM3C cytokine-like factors [85] and SP4, DOCK10, ITGB1, ITGA4 and RORA, five factors mentioned previously. Fifteen Cluster 3 GOIs contain TCF/LEF binding sites. This Wnt-regulated group includes three factors involved with mRNA maturation, RASA1, SFRS7, NUDT21, and three involved with protein modification or degradation, ZDHHC2, MGAT4A, and YOD1.

#### Cluster 4

These nine genes exhibit an immediate-early response, sharply decreasing in expression from baseline to BAC2, and then moderately increasing at BAC3 (Figure 2). Two of these genes have not been characterized (Additional file 3). The remaining seven genes form a single IPA network with functions including Cell Growth and Proliferation, and Small Molecule Biochemistry that contains TP53 and NF-κB as central non-GOIs. Within this cluster, the NF-κB inhibitor, NFKBIA, and the pro-inflammatory calcium-binding protein S100A12 are found highlighting the delicate interplay in regulatory control of NF-κB activity. Also in this cluster are MED6, a member of the Mediator complex [86], and PADI4, a gene that plays a role in granulocyte and macrophage development in inflammation and the immune response [87].

From BIOBASE, p38 MAPK is implicated in the regulation of Cluster 4 immediate-early response genes through binding sites for the JUN/ATF2 heterodimer in the PADI4 and S100A12 genes, and ELK1 binding to PHC2, NFKBIA, and MED6. In addition, BIOBASE also associated Cluster 4 GOIs PHC2, MED6, GPSM3, PADI4, NFKBIA with the apoptosis signaling pathway through binding matrices for ELK-1, FOXO3 and 4, and MYC (Table 4), of which four are shared with p38 MAPK signaling (Figure 3). PHC2 is highly expressed in whole blood and can associate with BMI1 a GOI factor in Cluster 2 associated with cell division in hematopoietic stem cells [88]. GPSM3 is unique in containing only a FOXO3 binding site.

#### Cluster 5

This is the largest expression cluster, containing 67 early response GOIs with a distinctive decrease in expression from BAC2 to BAC5 (Figure 2). Due to computational limitations in network size, IPA created two networks that could be merged. Examination of the merged network reveals central non-GOIs that include NF-κB, p38 MAPK, insulin, IL-1, -6, and −12, TNF, TGFB1, and d-glucose as an activation agent. The functions assigned to the individual networks include for Network 1: Immune Cell Trafficking, Hematological System Function, and Apoptosis and for Network 2: Cell Growth, Proliferation and Development, Gene Expression, and Carbohydrate Metabolism (Table 3).

Cluster 5 contains three members of the pentose phosphate pathway (PGLS, TALDO1, TKT), which is inhibited in the presence of ethanol [15]. GOI IMPA2 is important in phosphatidylinositol and insulin signaling [89]. Four members of the S100A calcium binding family are members of Cluster 5 and are network targets of TGFB1. Additionally in the network, S100A4 is regulated by the NF-κB complex, ERK and AP-1; S100A9 is regulated by P38 MAPK, and S100A6 is linked to activation of JNK, which in turn is integral to IL-1 and IL-12 signaling [20].

BIOBASE analysis linked 44 Cluster 5 genes to the p38 MAPK signaling pathway through binding sites for transcription factors ELK-1, CREB1, NF-κB, and SPI-1 (Figure 3). Most have binding sites for more than one of these factors. However, seventeen of the 44 contain only SPI-1 binding sites and function in defense response, the pentose phosphate shunt, inositol phosphate signaling, and S100A signaling.

#### Cluster 6

These four genes constitute a late-response cluster specific to falling ethanol levels with an expression spike at BAC4 and return to baseline at BAC5 (Figure 2). IPA put two of the four members, HLA-DQA1 and HLA-DQB1, the subunits of the DQ heterodimer and components of major histocompatibility complex CII [90] in an immune response network. GIMAP2 and MXRA7 were not assigned to a network. GIMAP2 is uniquely expressed in whole blood and T-cells [42], and is a GTPase in the immunity-associated protein family [91]. MXRA7 is a ubiquitously expressed gene with unknown function.

#### Cluster 7

These five genes show a delayed response, increasing in expression levels at BAC4 and 5 as ethanol levels decrease. Four of the five genes appear in an IPA network with gene expression as the top function. HMGB1(amphoterin), is a cytokine mediator of inflammation [92] through RAGE ([93], Figure 4). Also in this network are UBA6, regulated by TNF and INF-gamma [94], RGS18, a whole blood-specific [42] G-protein signaling attenuator [95], PPP4R2, involved in the maturation of splicosomal snRNPs [96]. EVI2A is an uncharacterized factor not included in the IPA network with blood-specific expression [42]. Cluster 7 members regulate a range of cellular mechanisms, including protein recycling [94], signal transduction [95], immuno-modulation [92], and transcript maturation [96].

BIOBASE analysis showed that HMGB1 carries binding sites for STAT1, 3 or 5A. STAT1 can form homo- or heterodimers [97] with STAT3, which is also upregulated by acute ethanol exposure as part of the Src pathway [26]. STAT5a, an antiapoptotic factor [98], shows cell-specific response to ethanol, is up-regulated in T cells and down-regulated in NK cells and induced by a number of cytokines [99]. HMGB1 is an antiapoptotic factor that binds RAGE to elicit release of cytokines [100,101].

### Summary

To identify potential gene expression markers and increase our understanding of the biological response to acute ethanol ingestion, we used a microarray and qRT-PCR-based approach on whole-blood RNA samples collected from human subjects administered orange juice with and without ethanol.

Our microarray data analysis revealed biases in the three analysis methods used. At the qRT-PCR-validated significance limits used, the LPE- and EDGE-lists share only one gene. Timecourse was more comprehensive; this list shared 13 genes with the LPE-derived list and 17 genes with the EDGE-derived list. This observation extended to the expression patterns detected upon clustering of the genes. The LPE t-test uniquely identified 42 of 44 genes in Cluster 2 and 38 of 47 Cluster 3 genes, whereas in Cluster 5, 39 of 67 genes were identified only by EDGE. All of the Cluster 4 and unclustered genes were from the Timecourse50 list. The explanation of the bias is likely rooted in the nature of the relative expression change patterns; for example, LPE was the analysis method that detected the majority of genes increasing in expression across the experimental time course. Most importantly all three methods identified genes that were confirmed by qRT-PCR as differentially expressed (Tables 1 and 2).

Certain biological regulatory motifs emerged from our analysis, key among them, calcium flux, the influence of NF-κB and p38 MAPK.

#### Calcium binding

There are 16 calcium binding genes in the ethanol GOIs, as characterized by DAVID (Additional file 3). Notably, six are members of the S100A family. This is a group of more than 25 small, acidic, EF-hand (calcium-binding) proteins. They can form homo- or heterodimers leading to complex calcium signaling dependent on the available S100A populations [102]. Four, S100A4, -6, -9, and −11, are found in Cluster 5, an early response cluster with decreased expression. S100A12 belongs to Cluster 4 and has an immediate early decreased expression profile. The unclustered S100A8 has an early decrease in expression but returns to baseline at BAC5. Taken together, the known decrease in NF-κB mediated inflammation from ethanol [20] could be the result of decreased S100A4, -6, -11, and −12 binding to the pro-inflammatory RAGE ([102,93], Figure 4).

Five calcium binding genes showed increased expression as members of the early response Cluster 2 and immediate early Cluster 3 (Figure 2). Cluster 2 includes TBC1D9, linked to hepatic disease [103], PDP1, a regulator of pyruvate levels, GALNT7, a member of the O-glycosylation pathway [104], and the mannose hydrolase MAN1A1. Cluster 3 contains ITGA4, an integrin highly expressed in blood and leukocytes linked to recruitment of leukocytes to sites of inflammation [105].

It is known that levels of ionized calcium in the blood decrease in the presence of acute ethanol exposure, and in rats, the level and duration of the decrease is correlated with the concentration of ethanol [106]. Decreased calcium would further modulate inflammation through decreased S100 protein oligomerization. It is known that in T-cell activation, ionized calcium regulates entry into the cell cycle through the induction of gene transcription by reversing Rb1 inhibition of E2F factors [107]. We found that E2F factors were linked to Cluster 3 expression (Table 4) suggesting that the known ethanol-dependent reduction of ionized calcium levels [106] is acting at the transcription level to inhibit, then release entry into the cell-cycle.

#### NF-κB as central regulator

NF-κB is a central regulator appearing in all but one of the cluster function analyses (Figure 4), indicating a level of co-regulation linking ethanol ingestion to innate immunity and the inflammatory response. Combining temporal expression patterns with direct and indirect molecular interactions defined by pathway analysis, we hypothesize that NF-κB-regulated inflammation is down-regulated early and moderates as blood ethanol levels decrease. The decreasing inflammatory response results from lower levels of S100A proteins and their effect on RAGE induction of NF-κB, and RORA activation of NFKBIA. BMI1 is induced in an immediate early response and may counter the decreased inflammatory response. Evidence for the return to normal levels of NF-κB activity is in the late increase in expression of S100A8 and Cluster 1 genes KLF3, UBE2D3, and PF4V1, and may be a result of increased expression of Cluster 7 gene, HMGB1 (Figure 4) and subsequent activation of RAGE (reviewed in [108]). Modulating the late increase in NF-κB activity is BAX, another Cluster 1 gene. NF-κB acts as both an enhancer and suppressor in the MHC CII promoter [109], suggesting NF-κB regulation of the expression spike in the two Cluster 6 MHC CII components.

#### Signaling pathway membership

BIOBASE is a powerful tool that complements the IPA analysis by looking for conserved transcription factor binding sites in the GOIs and moving upstream to identify significant signaling pathways. Within a 1200 bp window, several transcription factors were found to bind to GOI promoters across more than one cluster. SPI-1, ATF2, and CREB1 are each binding within genes in multiple but not the same clusters, (Table 4A). ATF2 and JUN are both found in Clusters 3 and 4, which have differing expression patterns overall, but share a marked decrease in expression from BAC1 to BAC2. The shared ATF2/CREB binding site is found in Clusters 2 and 3 which have similar expression patterns, especially from BAC2 to BAC5.

The p38 MAPK pathway was identified as a central regulatory pathway for clusters 1, 2, 3, 4, and 5 (Figure 3). These clusters have diverse expression patterns likely reflecting the heterogeneity of cells in whole blood, interactions with various other signaling molecules or transcription factors, and the variability of post-translational modification, none of which can be detected by BIOBASE. The p38-regulated functional categories predicted by BIOBASE include the innate and inflammatory immune responses, ubiquitination, apoptosis, and energy metabolism.

Unique signaling pathways that may modulate p38 MAPK were predicted by BIOBASE within three clusters (Figure 3). GOIs within Cluster 2 contain binding sites for IRF3 and IRF7, which are linked to TLR4 signaling. TLR4 signaling activates the p38 MAPK pathway and is especially sensitive to ethanol exposure, exhibiting a dose-dependent response curve [110].

Binding sites for ETF2 are found within 12 genes in Cluster 3; these GOIs are involved in ubiquitination and cytokine production. The binding site for LEF/TCF is found within 13 GOIs in Cluster 3, in which are also found binding sites for transcription factors from multiple signaling pathways that regulate hemoglobin biosynthesis and immune response regulation. The apoptosis network regulated through FOXO3 and 4, and MYC binding, in concert with p38 MAPK signaling through ELK1, is unique to Cluster4. Unlinked to p38 MAPK signaling is STAT signaling found in the delayed response Cluster 7 (Table 4B) and known to be up-regulated in response to acute ethanol [19,26].

#### Biomarker identification

Conceptually, markers for ethanol consumption could be of two types, actual BAC, or impairment. Expression patterns of the clustered genes neither positively nor negatively correlate to ethanol concentration; however, two unclustered genes correlate to BAC more closely, CRISPLD2 and NUDT4. These two genes have opposite patterns of expression exhibiting their most extreme variation from baseline to 0.08% BAC and returning to baseline expression at 0.02% BAC, the last collection point in this study and are potentially useful markers for BAC.

For forensic toxicology, impairment is the least understood but most important metric. Here, we have shown that most of our 203 genes do not return to baseline at 0.02% BAC, suggesting that further efforts should concentrate on correlating cognition to expression patterns, thereby capturing the “hangover” effect. Specifically, several genes in Clusters 2 such as PDP1, GNAQ and TGFBR1, and Cluster 5 members like S100A4, -6, and −8 and the three pentose phosphate shunt members TALDO1, TKT and PGLS, exhibit expression patterns consistently increasing or decreasing over the entire experimental time-course, suggesting that they may return to baseline as cognition recovers. Further effort to determine at what cognitive level they returned to baseline would be informative. Likewise, the delayed response genes in Cluster 7 (Figure 2) may be indicative of cognitive impairment.

## Conclusions

We determined patterns of gene expression related to acute exposure to ethanol. Our analysis suggests that we could detect significant gene expression changes related to imbibed ethanol using RNA isolated from blood. We found that members of each cluster were linked by common biological processes, signaling pathways, and functions including:

– protein synthesis and modification

– hematological and immune functions, especially innate immunity

– inflammation

– p38 MAPK and NF-κB signaling

central metabolism and small molecule metabolism

Additionally, our findings support the workflow described here for selecting candidate biomarker genes for future studies. Those studies would correlate performance limitations to ETOH-dependent gene expression patterns and, using currently available toxicological tests to monitor BAC, must extend beyond the breathalyzer limit of detection of 0.02%, to capture cognitive “hangover effects”. A gene expression-based test for ingested ethanol would provide additional sensitivity in those forensic instances where standard toxicological tests cannot discriminate between ingestion and post-mortem ethanol sources. We further expect that understanding gene expression changes and their correlation to human performance metrics will facilitate modification of flight regulations based on increased understanding of ethanol’s impact on performance independent of blood alcohol concentration.

## Abbreviations

BAC: Blood alcohol concentration; CAMI: Civil aerospace medical institute; Ct: Cycle threshold; DWI: Driving while intoxicated; g/L: Grams per liter; g/dL: Grams per deciliter; GOI: Gene of interest; GOIs: Genes of interest; 5-HTOL: 5-hydroxytryptophol; 5-HIAA: 5-hydroxyindole-3-acetic acid; OJ: Orange juice control; qRT-PCR: Quantitative polymerase chain reaction; ROS: Reactive oxygen species; RIN: RNA integrity number; TFBS: Transcription factor binding site.

## Competing interests

The authors declare that they have no competing interests.

## Authors’ contributions

DMK performed qRT-PCR, and carried out analysis of GOIs. VW collected blood samples, performed RNA isolation and microarray hybridization. DLS and DC recruited subjects and collected blood samples. DB recruited subjects, performed informatics analysis, and supervised the study. DMK and DB wrote and reviewed the manuscript. All authors read and approved the final manuscript.

## Pre-publication history

The pre-publication history for this paper can be accessed here:

http://www.biomedcentral.com/1755-8794/6/26/prepub

## Supplementary Material

Additional file 1 Total RNA yield and integrity. Click here for file

Additional file 2 Primers used in study. Click here for file

Additional file 3 K means expression cluster members. Click here for file

## References

[B1] Li G Baker SP Qiang Y Robok GW McCarthy ML Alcohol violations and aviation accidents: findings from the U. S. mandatory alcohol testing program Aviation Space Environ Med 2007 78 5 510 513 PMC2041869 17539446

[B2] Satter RG 2009 New York: Associated Press

[B3] Pow H The Daily Mail 2013 London: Daily Mail and General Trust

[B4] Morrow D Yesavage J Leirer V Dolhert N Taylor J Tinklenberg J The time-course of alcohol impairment of general aviation pilot performance in a Frasca 141 simulator Aviat Space Environ Med 1993 64 8 697 705 8368982

[B5] Newman DG Alcohol and human performance from an aviation perspective: a review 2004 Canberra: Australian Transport Safety Bureau

[B6] Taylor JL Dolhert N Morrow D Friedman L Yesavage JA Acute and 8-hour effects of alcohol (0.08% BAC) on younger and older pilots’ simulator performance Aviat Space Environ Med 1994 65 718 725 7980331

[B7] NTSB Review of U.S. Civil Aviation Accidents 2007–2009 2011 Washington, D.C: National Transportation and Safety Board

[B8] McFadden KL DWI convictions linked to a higher risk of alcohol-related aircraft accidents Hum Factors 2002 44 4 522 529 10.1518/0018720024496962 12691362

[B9] Canfield DV Hordinsky J Millett DP Endecott B Smith D Prevalence of drugs and alcohol in fatal civil aviation accidents between, between 1994 and 1998 Aviat Space Environ Med 2001 72 2 120 124 11211040

[B10] Li G Baker SP Lamb MW Qiang Y McCarthy ML Characteristics of alcohol-related fatal general aviation crashes Acc Anal Prev 2005 37 143 148 10.1016/j.aap.2004.03.005 15607285

[B11] Botch SR Johnson RD Alcohol-related aviation accidents involving pilots with previous alcohol offenses Off Aerospace Med Rep 2008 08 22 1 8

[B12] Helander A Beck O Jones AW Urinary 5HTOL/5HIAA as biochemical marker of postmortem ethanol synthesis Lancet 1992 340 1159 1279333 10.1016/0140-6736(92)93184-o

[B13] Johnson RD Lewis RJ Canfield DV Blank CL Accurate assignment of ethanol origin in postmortem urine: liquid chromatographic-mas spectrometric determination of serotonin metabolites J Chromatography B Analyt Technol Biomed Life Sci 2004 805 223 224 10.1016/j.jchromb.2004.02.042 15135094

[B14] McMonagle J Felig P Effects of ethanol ingestion on glucose tolerance Metab Clin Experimen 1975 24 5 625 632 10.1016/0026-0495(75)90142-0 1128232

[B15] Badawy AA-B A review of the effects of alcohol on carbohydrate metabolism Brit J Alcohol Alcoholism 1977 12 3 120 136

[B16] Wu D Cederbaum AJ Alcohol, oxidative stress, and free radical damage Alcohol Res Health 2003 27 4 277 284 15540798 PMC6668865

[B17] Arbabi S Garcia I Bauer GJ Maier RV Alcohol (Ethanol) inhibits IL-8 and TNF: Role of the p38 pathway J Immunol 1999 162 7441 7445 10358198

[B18] Xiao C Ghosh S NF-kB as evolutionarily conserved mediator of immune and inflammatory responses Advan Exp Med Biol 2005 560 41 45 10.1007/0-387-24180-9_5 15932018

[B19] Mandrekar P Catalano D White B Szabo G Moderate alcohol intake in humans attenuates monocyte inflammatory responses: inhibition of nuclear regulatory factor Kappa B and induction of interleukin 10 Alcohol Clin Exp Res 2006 30 1 135 139 10.1111/j.1530-0277.2006.00012.x 16433741

[B20] Szabo G Mandrekar P Oak S Mayerle J Effect of ethanol on inflammatory responses Pacreatology 2007 7 115 123 10.1159/000104236 PMC2790780 17592223

[B21] Brancho D Tanaka N Jaeschke A Ventura J-J Kelkar N Tanaka Y Kyuuma M Takeshita T Flavell RA Davis RJ Mechanism of p38 MAP kinase activation in vivo Genes Dev 2003 17 1969 1978 10.1101/gad.1107303 12893778 PMC196252

[B22] Mandrekar P Bala S Catalano D Kodys K Szabo G The opposite effects of acute and chronic alcohol on lipopolysaccharide-induced inflammation are linked to IRAK-M in human monocytes J Immunol 2009 183 1320 1327 10.4049/jimmunol.0803206 19561104 PMC3845821

[B23] Saha RN Jana M Pahan K MAPK p38 regulates transcriptional activity of NF-κB in primary human astrocytes via acetylation of p65 J Immunol 2007 179 7101 7109 17982102 10.4049/jimmunol.179.10.7101 PMC2701546

[B24] Mattson MP Chan SL Calcium orchestrates apoptosis Nature Cell Biol 2003 5 12 1041 1043 10.1038/ncb1203-1041 14647298

[B25] Pastorino JG Shulga N Hoek JB TNF-alpha-induced cell death in ethanol-exposed cells depends on p38 MAPK signaling but is independent of Bid and caspase-8 Am J Physiol Gastrointest Liver Physiol 2003 285 G503 G516 12748063 10.1152/ajpgi.00442.2002

[B26] Norkina O Dolganiuc A Shapiro T Kodys K Mandraker P Szabo G Acute alcohol activates STAT3, AP-1, and Sp-1 transcription factors via the family of Src kinases to promote IL-10 production in human monocytes J Leukocyte Biol 2007 82 3 752 762 10.1189/jlb.0207099 17575268

[B27] Norkina O Dolganiuc A Catalano D Kodys K Mandraker P Syed A Efros M Szabo G Acute alcohol intake induces SOCS1 and SOCS3 and inhibits cytokine-induced STAT1 and STAT3 signaling in human monocytes Alcohol Clin Exp Res 2008 32 9 1565 1573 10.1111/j.1530-0277.2008.00726.x 18616672 PMC4116614

[B28] Szabo G Catalano D White B Mandrekar P Acute alcohol consumption inhibits accessory cell function of monocytes and dendritic cells Alcohol Clin Exp Res 2004 28 5 824 828 10.1097/01.ALC.0000127104.80398.9B 15166660

[B29] Jones AW Pounder DJ Karch SB Update on clinical and forensic analysis of alcohol Drug abuse handbook 2007 2 Boca Raton: CRC Press 333 376

[B30] Strayer DL Drews FA Crouch DJ A comparison of the cell phone driver and the drunk driver Hum Factors 2006 48 2 381 391 10.1518/001872006777724471 16884056

[B31] Vu NT Zhu H OE D Huggins ME White VL Chaturvedi AK Canfield DV Whinnery JE Isolation of RNA from peripheral blood cells: a validation study for molecular diagnostics by microarray and kinetic RT-PCR assays-Application in aerospace medicine Office Aerospace Medi Rep 2004 04 01 1 12

[B32] Irizarry RA Hobbs B Collin F Beazer-Barclay YD Antonellis KJ Scherf U Speed TP Exploration, normalization, and summaries of high density oligonucleotide array probe level data Biostatistics 2003 4 2 249 264 10.1093/biostatistics/4.2.249 12925520

[B33] Wu Z Irizarry R Gentleman R Murillo F Spencer F A model-based background adjustment for oligonucleotide expression arrays J Am Stat Assoc 2004 99 909 917 10.1198/016214504000000683

[B34] Jain N Thatte J Braciale T Ley K O’Connell M Lee JK Local-pooled-error test for identifying differentially expressed genes with a small number of replicated microarrays Bioinformatics 2003 19 15 1945 1951 10.1093/bioinformatics/btg264 14555628

[B35] Benjamini Y Hochberg Y Controlling the false discovery rate: a practical and powerful approach to multiple testing J Royal Stat Soc Series B (Methodological) 1995 57 1 289 300

[B36] Leek JT Monsen E Dabney AR Storey JD EDGE: extraction and analysis of differential gene expression Bioinformatics 2006 22 4 507 508 10.1093/bioinformatics/btk005 16357033

[B37] Tai YC Speed TP A multivariate empirical Bayes statistic for replicated microarray time course data Ann Stat 2006 34 5 2387 2412 10.1214/009053606000000759

[B38] Bioconductorhttp://www.bioconductor.org/. Accessed July 20, 2013

[B39] Futschik ME Carlisle B Noise-robust soft vlustering of gene expression time-course data J Bioinform Comput Bioil 2005 3 4 965 988 10.1142/S0219720005001375 16078370

[B40] Dennis G Jr Sherman BT Hosack DA Yang J Gao W Lane HC Lempicki RA DAVID: database for annotation, visualization, and integrated discovery Genome Biol 2003 4 epub PMC193660 12734009

[B41] Wu C Orozco C Boyer J Leglise M Goodale J Batalov S Hodge CL Haase J Janes J Huss JW III BioGPS: an extensible and customizable portal for querying and organizing gene annotation resources Genome Biol 2009 10 10 R130 19919682 10.1186/gb-2009-10-11-r130 PMC3091323

[B42] BioGPShttp://biogps.org/#goto=welcome. Accessed 07.18.13

[B43] BIOBASEhttp://www.biobase-international.com. Accessed 07.18.13

[B44] Kel A Voss N Valeev T Stegmaier P Kel-Margoulis O Wingerder E ExPlain: finding upstream drug targets in disease gene regulatory networks SAR QSAR Environ Res 2008 19 5–6 481 494 18853298 10.1080/10629360802083806

[B45] Edgar R Domrachev M Lash AE Gene expression omnibus: NCBI gene expression and hybridization array data repository Nucleic Acids Res 2002 30 1 207 210 10.1093/nar/30.1.207 11752295 PMC99122

[B46] Andersen CL Jensen JL Orntoft TF Normalization of real-time quantitative reverse transcription-PCR data: a model-based variance estimation approach to identify genes suited for normallization, applied to bladder and colon cancer data sets Cancer Res 2004 64 5245 5250 10.1158/0008-5472.CAN-04-0496 15289330

[B47] Vandesompele J De Preter K Pattyn F Poppe B Van Roy N De Paepe A Speleman F Accurate normalization of real-time quantitative RT-PCR data by geometric averaging of multiple internal control genes Genome Biol 2002 3 7 1 12 epub 10.1186/gb-2002-3-7-research0034 PMC126239 12184808

[B48] Pfaffl MW Horgan GW Dempfle L Relative expression software tool (REST^c^) for group-wise comparison and statistical analysis of relative expression results in real-time PCR Nucleic Acids Res 2002 30 9 e36 10.1093/nar/30.9.e36 11972351 PMC113859

[B49] Fenner BJ Scannell M Prehn JH Identification of polyubiquitin binding proteins involved in NF_kappaB signaling using protein arrays Biochim Biophys Acta 2009 1794 7 1010 1016 10.1016/j.bbapap.2009.02.013 19285159

[B50] Parikh N Sade H Kurian L Sarin A The Bax N terminus is required for negative regulation by the mitogen-activated protein kinase kinase and Akt signaling pathways in T cells J Immunol 2004 173 6220 6227 15528359 10.4049/jimmunol.173.10.6220

[B51] Cojocaru M Bouchard A Cloutier P Cooper JJ Varzavand K Price DH Coulombe B Transcription factor IIS cooperates with the E3 ligase UBR5 to ubiquitinate the CDK9 subunit of the positive transcription elongation factor B J Biol Chem 2011 286 7 5012 5022 10.1074/jbc.M110.176628 21127351 PMC3037613

[B52] Turner J Crossley M Basic Kruppel-like factor functions within a network of interacting haematopoietic transcription factors Int J Biochem Cell Biol 1999 31 10 1169 1174 10.1016/S1357-2725(99)00067-9 10582345

[B53] Saville MK Sparks A Xirodimas DP Julie W Stevenson LF Jean-Christophe B Woods YL Lane DP Regulation of p53 by the ubiquitin-conjugating enzymes UbcH5B/C in vivo J Biol Chem 2004 279 40 42169 42181 10.1074/jbc.M403362200 15280377

[B54] Nathan C Ding A Nonresolving inflammation Cell 2010 140 6 871 882 10.1016/j.cell.2010.02.029 20303877

[B55] Arruda LB Sim D Chikhlilkar PR Maciel M Akasaki K August T Marques ET Dendritic cell-lysosomal-associated membrane protein (LAMP) and LAMP-1-HIV-1 Gag chimeras have distinct cellular trafficking pathways and prime T and B cell responses to a diverse repertoire of epitopes J Immunol 2006 177 2265 2275 16887987 10.4049/jimmunol.177.4.2265

[B56] Welch MD DePace AH Verma S Iwamatsu A Mitchison TJ The human Arp2/3 complex is composed of evolutionarily conserved subunits and is locallized to cellular regions of dynamic actin filament assembly J Cell Biol 1997 138 2 375 384 10.1083/jcb.138.2.375 9230079 PMC2138188

[B57] Akashi-Takamura S Miyake K TLR accessory molecules Curr Opin Immunol 2008 20 4 420 425 10.1016/j.coi.2008.07.001 18625310

[B58] von Hundelshausen P Petersen F Brandt E Platelet-derived chemokines in vascular biology J Thrombosis Haemostasis 2007 97 5 704 713 10.1160/th07-01-0066 17479180

[B59] Kokura K Sun L Bedford MT Fang J Methyl-H3K9-binding protein MPP8 mediates E-cadherin gene silencing and promotes tumour cell motility and invasion Eur Mole Biol Org J 2010 29 21 3673 3687 10.1038/emboj.2010.239 PMC2982762 20871592

[B60] Wontakal SN Guo X Smith C MacCarthy T Bresnick EH Bergman A Snyder MP Weissman SM Zheng D Skoultchi AI A core erythroid transcriptional network is repressed by a master regulator of myelo-lymphoid differentiation Proc Nat Acad Sci 2012 109 10 3832 3837 10.1073/pnas.1121019109 22357756 PMC3309740

[B61] Yordy JS Muise-Helmericks RC Signal transduction and the Ets family of transcription factors Oncogene 2000 19 6503 6513 10.1038/sj.onc.1204036 11175366

[B62] Piccinini M Mostert M Alberto G Ramondetti C Novi RF Dalmasso P Rinaudo MT Down-regulation of pyruvate dehydrogenase phosphatase in obese subjects is a defect that signals insulin resistance Obesity Res 2005 13 4 678 686 10.1038/oby.2005.76 15897476

[B63] Ikonomov OC Sbrissa D Mlak K Shisheva A Requirement for PIKfyve enzymatic activity in acute and long-term insulin cellular effects Endocrinology 2002 143 12 4742 4754 10.1210/en.2002-220615 12446602

[B64] Park I-K Morrison SJ Clarke MF Bmi1, stem cells, and senescence regulation J Clin Invest 2004 113 2 175 179 14722607 10.1172/JCI20800 PMC311443

[B65] Ame JC Spenlehauer C de Murica G The PARP superfamily Bioessays 2004 26 8 882 893 10.1002/bies.20085 15273990

[B66] Damcott CM Hoppman N Ott SH Reinhart LJ Wang J Pollin TI O’Connell JR Mitchell BD Shuldiner AR Polymorphisms in both promoters of hepatocyte nuclear factor 4-A are associated with type 2 diabetes in the Amish Diabetes 2004 53 3337 10.2337/diabetes.53.12.3337 15561969

[B67] Richards MW O’Regan L Mas-Droux C Blot JM Cheung J Hoelder S Fry AM Bayliss R An autoinhibotory tyrosine motif in the cell-cycle-regulated Nek7 kinase is relesed through binding of Nek9 Mol Cell 2009 36 4 560 570 10.1016/j.molcel.2009.09.038 19941817 PMC2807034

[B68] Yellaturu CR Deng X Cagen LM Wilcox HG Mansbach CM II Siddiqui SA Park EA Raghow R Elam MB Insulin enhances post-translational processing of nascent SREBP-1c by promoting its phosphorylation and association with COP11 vesicles J Biol Chem 2009 284 12 7518 7532 10.1074/jbc.M805746200 19158095 PMC2658047

[B69] Takatsu H Baba K Shima T Hiroyuki U Kato U Umeda M Nakayama K Shimn H-W ATP9B, a P4-ATPase (a putative aminophospholipid translocase), localizes to the trans-Golgi network in a CDC50 protein-independent manner J Biol Chem 2011 286 44 38159 38167 10.1074/jbc.M111.281006 21914794 PMC3207472

[B70] Ouwens DM de Ruiter ND van der Zon GCM Carter AP Schouten J van der Burgt C Kooistra K Bos JL Maassen JA van Dam H Growth factors can activate ATF2 via a two-step mechanism: phosphorylation of Thr71 through the Ras-MEK-ERK pathway and of Thr69 through RalGDS-Src-p38 Eur Mole Biol Org J 2002 21 14 3782 3793 10.1093/emboj/cdf361 PMC126107 12110590

[B71] Gee K Angel JB Mishra S Blahoianu MA Kumar A IL-10 regulation by HIV-Tat in primary human monocytic cells: involvement of calmodulin/calmodulin-dependent protein kinase-activated p38 MAPK and sp-1 and CREB-1 transcription factors J Immunol 2007 178 798 807 17202341 10.4049/jimmunol.178.2.798

[B72] Tomar A Lim S-T Lim Y Schlaepfer DD A FAK-p120RasGAP-p190RhoGAP complex regulates polarity in migrating cells J Cell Sci 2009 122 11 1852 1862 10.1242/jcs.046870 19435801 PMC2684836

[B73] Zhu Y McAvoy S Kuhn R Smith DI RORA, a large common fragile site gene, is involved in cellular stress response Oncogene 2006 25 2901 2908 10.1038/sj.onc.1209314 16462772

[B74] Hammarsund M Wilson W Corcoran M Merup M Einhorn S Grander D Sangfelt O Identification and characterization of two novel human mitochondrial elongation factor genes, hEFG2 and hEFG1, phylogenetically conserved through evolution Human Gen 2001 109 542 550 10.1007/s00439-001-0610-5 11735030

[B75] Lignitto L Carlucci A Sepe M Stefan E Cuomo O Nistico R Scorziello A Savoia C Garbi C Annunziato L Control of PKA stability and signalling by the RING ligase praja2 Nat Cell Biol 2011 13 4 412 422 10.1038/ncb2209 21423175

[B76] Aplin AE Howe A Alahari SK Juliano RL Signal transduction and signal modulation by cell adhesion receptors: the role of integrins, cadherins, immunoglobulin-cell adhesion molecules, and selectins Pharmacol Rev 1998 50 2 197 262 9647866

[B77] Zhao Y Zhou L Liu B Deng Y Wang Y Wang Y Huang W Yuan W Wang Z Zhu C ZNF325, a novel human zinc finger protein with a RBaK-like RB-binding domain, inhibits AP-1- and SRE-mediated transcriptional activity Biochem Biophysic Res Comm 2006 346 1191 1199 10.1016/j.bbrc.2006.06.031 16806083

[B78] Hallier M Tavitian A Moreau-Gachelinss F The transcription factor Spi-1/PU.1 binds RNA and interferes with the RNA-binding protein p54nrb* J Biol Chem 1996 271 19 11177 11181 10.1074/jbc.271.19.11177 8626664

[B79] Yelo E Bernardo MV Gimeno L Alcaraz-Garcia MJ Majado MJ Parrado A Dock10, a novel CZH protein selectively induced by interleukin-4 in human B lymphocytes Mole Immunol 2008 45 12 3411 3418 10.1016/j.molimm.2008.04.003 18499258

[B80] Jutooru I Chadalapaka G Lei P Safe S Inhibition of NF-κB and pancreatic cancer cell and tumor growth by curcumin is dependent on specificity protein down-regulation J Biol Chem 2012 285 33 25332 25344 20538607 10.1074/jbc.M109.095240 PMC2919096

[B81] Dailey HA Sellers VM Dailey TA Mammalian ferrochelatase J Biol Chem 1994 269 1 390 395 8276824

[B82] Ho TH Charlet-B M Poulos MG Siingh G Swanson MS Cooper TA Muscleblind proteins regulate alternative splicing Eur Mole Biol Org J 2004 23 3103 3112 10.1038/sj.emboj.7600300 PMC514918 15257297

[B83] Jeong SM Lee C Lee SK Kim J Seong RH The SWI/SNF chromatin-remodeling complex modulates peripheral T cell activation and proliferation by controlling AP-1 expression J Biol Chem 2010 285 4 2340 2350 10.1074/jbc.M109.026997 19910461 PMC2807292

[B84] Matthews SA Dayalu R Thompson LJ Scharenberg AM Regulation of protein kinase Cv by the B-cell antigen receptor J Biol Chem 2003 278 11 9086 9091 10.1074/jbc.M211295200 12506120

[B85] Zhu Y Xu G Patel A McLaughlin MM Silverman C Knecht KSS Li X McDonnell P Mirabile R Cloning, expression, and initial characterization of a novel cytokine-like gene family Genomics 2002 80 2 144 150 10.1006/geno.2002.6816 12160727

[B86] Kim S Xu X Hecht A Boyer TG Mediator is a transducer of Wnt/B-catenin signaling J Biol Chem 2006 281 20 14066 14075 10.1074/jbc.M602696200 16565090

[B87] Chang X Yamada R Suzuki A Sawada T Yoshino S Tokuhiro S Yamamoto K Localization of peptidylarginine deiminase 4 (PADI4) and citrullinated protein in synovial tissue of rheumatoid arthritis Rheumatology 2005 44 1 40 50 10.1093/rheumatology/keh414 15466895

[B88] Gunster MJ Satijn DP Hamer KM den Blaauwen JL de Bruijn D Alkema MJ van Lohuizen M van Driel R Otte AP Identification and characterization of interactions between the vertebrate polycomb-group protein BMI1 and human homologs of polyhomeotic Mole Cell Biol 1997 17 4 2326 2335 10.1128/mcb.17.4.2326 PMC232081 9121482

[B89] Ohnishi T Ohba H Seo K-C Im J Sato Y Iwayama Y Furuichi T Chung S-K Takeo Y Spatial expression patterns and biochemical properties distinguish a second myo-inositol monophosphatase IMPA2 from IMPA1 J Biol Chem 2007 282 1 637 646 17068342 10.1074/jbc.M604474200

[B90] Kaufman JF Auffray C Korman AJ Shackelford DA Strominger J The class II molecules of the human and murine major histocompatibility complex Cell 1984 36 1 1 13 10.1016/0092-8674(84)90068-0 6198089

[B91] Krucken J Schroetel R Muller I Saidani N Marinovski P Benten W Stamm O Underlich F Coomparative analysis of the human gimap gene cluster encoding a novel GTPase family Gene 2004 341 291 304 15474311 10.1016/j.gene.2004.07.005

[B92] Wang H Bloom O Zhang M Ombrellino M Che J Frazier A Yang H Ivanova S Borovikova L Manogue KR HMG-1 as a late mediator of endotoxin lethality in mice Science 1999 285 5425 248 251 10.1126/science.285.5425.248 10398600

[B93] Leclerc E Fritz G Vetter SW Heizmann CW Binding of S100 proteins to RAGE: an update Biochim Biophys Acta 2009 1793 993 1007 10.1016/j.bbamcr.2008.11.016 19121341

[B94] Chiu Y-H Sun Q Chen ZJ E1-L2 activates both ubiquitin and FAT10 Mol Cell 2007 27 1014 1023 10.1016/j.molcel.2007.08.020 17889673

[B95] Yowe D Weich N Prabhudas M Poisson L Errada P Kapeller R Yu K Faron L Shen M Cleary J RGS18 is a myeloerythroid lineage-specific regulator of G-protein-signalling molecule highly expressed in megakaryocytes Biochem J (England) 2001 359 Pt 1 109 118 11563974 10.1042/0264-6021:3590109 PMC1222126

[B96] Carnegie GK Sleeman JE Morrice N Hastie CJ Peggie MW Philp A Lamond AI Cohen PT Protein phosphatase 4 interacts with the Survival of Motor Neurons complex and enhances the temporal localisation of snRNPs J Cell Sci 2003 116 Pt 10 1905 1913 12668731 10.1242/jcs.00409

[B97] Pensa S Regis G Boselli D Novelli F Poli V Stephanou A STAT1 and STAT3 in tumorigenesis: two sides of the same coin? JAK-STAT Pathway in Disease 2009 Austin: Landes Bioscience 100 121

[B98] Debierre-Grockiego F Anti-apoptotic role of STAT5 in haematopoietic cells and in the pathogenesis of malignancies Apoptosis 2004 9 6 717 728 15505414 10.1023/B:APPT.0000045785.65546.a2

[B99] Guo TL Zhang LX Chen JP Nguyen VA White KL Gao B Differential STAT5 activation and phenotypic marker expression by immune cells following low levels of ethanol consumption in mice Immunopharmacol Immunotoxicol 2002 24 1 121 138 10.1081/IPH-120003408 12022440

[B100] Muller S Ronfani L Bianchi ME Regulated expression and subcellular localization of HMGB1, a chromatin protein with a cytokine function J Int Med 2004 255 3 332 343 10.1111/j.1365-2796.2003.01296.x 14871457

[B101] Kokkola R Andersson A Mullins G Ostberg T Treutiger CJ Arnold B Nawroth P Andersson U Harris RA Harris HE RAGE is the major receptor for the proinflammatory activity of HMGB1 in rodent macrophages Scand J Immunol 2005 61 1 1 9 10.1111/j.0300-9475.2005.01534.x 15644117

[B102] Donato R S100: a multigenic family of calcium-modulated proteins of the EF-hand type with intracellualr and extracellular fruntional roles Int J Biochem Cell Biol 2001 33 7 637 668 10.1016/S1357-2725(01)00046-2 11390274

[B103] Ros J Libbrecht L Geuken M Jansen P Roskams T High expression of MDR1, MRP1, and MRP3 in the hapatic progenitor cell compartment and hepatocytes in severe human liver disease J Pathol 2003 200 5 553 560 10.1002/path.1379 12898590

[B104] Bennett EP Hassan H Hollingsworth MA Clausen H A novel human UDP-N-acetyl-D-galactosamine:polypeptide N-acetylgalactosaminyltransferase, GalNAc-T7, with specificity for partial GalNAc-glycosylated acceptor substrates FEBS Lett 1999 460 2 226 230 10.1016/S0014-5793(99)01268-5 10544240

[B105] Kummer C Petrich BG Rose DM Ginsberg MH A small molecule that inhibits the interaction of paxillin and alpha4 integrin inhibits accumulation of mononuclear leukocytes at a site of inflammation J Biol Chem 2010 285 13 9462 9469 10.1074/jbc.M109.066993 20097761 PMC2843196

[B106] Keiver K Duggal S Simpson ME Ethanol administration results in a prolonged decrease in blood ionized calcium levels in the rat Alcohol 2005 37 173 178 10.1016/j.alcohol.2005.07.008 16713506

[B107] Machaca K Ca2+ signaling, genes and the cell cycle Cell Calcium 2010 48 243 250 10.1016/j.ceca.2010.10.003 21084120 PMC3752338

[B108] Sparvero LJ Asafu-Adjei D Kang R Tang D Amin N Im J Rutledge R Lin B Amoscato AA Zeh HJ RAGE (Receptor for Advanced Glycation Endproducts), RAGE Ligands and their role in cancer and inflammation J Trans Med 2009 7 17 1 21 10.1186/1479-5876-7-17 PMC2666642 19292913

[B109] Brown AM Linhoff MW Stein B Wright KL Baldwin AS Basta PV Ting JP-Y Function of NF-κB/rel binding sites in the major histocompatibility complex class II invariant chain promoter is dependent on cell-specific binding of different NF-κB/rel subunits Mole Cell Biol 1994 14 5 2926 2935 10.1128/mcb.14.5.2926-2935.1994 PMC358660 8164652

[B110] Fernandez-Lizarbe S Pascual M Gascon MS Blanco A Guerri C Lipid rafts regulate ethanol-induced activation of TLR4 signaling in murine macrophages Mole Immunol 2008 45 7 2007 2016 10.1016/j.molimm.2007.10.025 18061674

