# Supplementary material for: Microarray characterization of gene expression changes in blood during acute ethanol exposure
Source: BMC Med Genomics. 2013 Jul 25;6:26. doi: 10.1186/1755-8794-6-26 (PMC3750403; doi:10.1186/1755-8794-6-26)
Supplement: Additional file 1 — Total RNA yield and integrity. [file 1755-8794-6-26-S1.pdf]

Additional File 1. Total RNA yield and integrity			
**Sample**	**260/280**	**Yield**	**RIN**
	**Ratio**	**ug**	**Range 1-10**
**Experimental**			
S1 - BAC1	3.76	7.31	7.6
S1 - BAC2	1.90	12.46	7.2
S1 - BAC3	1.97	15.50	7.7
S1 - BAC4	2.02	14.28	7.4
S1 - BAC5	2.18	10.60	7.6
S5 - BAC1	1.84	8.15	7.5
S5 - BAC2	1.98	7.18	8.4
S5 - BAC3	1.89	8.32	8.7
S5 - BAC4	2.51	5.20	8.7
S5 - BAC5	1.92	9.42	8.4
S8 - BAC1	1.81	7.18	7.5
S8 - BAC2	1.62	5.07	8.7
S8 - BAC3	1.57	4.08	7.9
S8 - BAC4	1.61	1.58	8.8
S8 - BAC5	1.49	3.41	8.0
S10 - BAC1	1.93	8.41	7.5
S10 - BAC2	1.85	9.26	8.3
S10 - BAC3	1.76	5.95	7.5
S10 - BAC4	1.85	4.75	8.1
S10 - BAC5	1.88	9.33	8.0
S13 - BAC1	1.58	10.22	8.1
S13 - BAC2	1.96	9.21	8.5
S13 - BAC3	1.95	11.02	8.1
S13 - BAC4	1.69	7.73	9.1
S13 - BAC5	2.08	3.17	9.0
S15 - BAC1	1.90	10.95	8.3
S15 - BAC2	1.93	8.24	9.2
S15 - BAC3	2.00	9.22	8.2
S15 - BAC4	1.90	5.14	8.7
S15 - BAC5	1.87	8.69	9.3
S17 - BAC1	1.91	8.87	8.3
S17 - BAC2	1.87	6.20	8.5
S17 - BAC3	2.36	8.91	8.6
S17 - BAC4	2.02	6.12	8.7
S17 - BAC5	2.00	16.40	9.3
S18 - BAC1	2.08	13.59	8.2
S18 - BAC2	2.09	12.71	8.3
S18 - BAC3	2.15	14.75	8.8
S18 - BAC4	2.36	9.47	8.8
S18 - BAC5	2.79	15.31	8.5

S19 - BAC1	2.02	20.56	8.4
S19 - BAC2	2.05	14.33	8.4
S19 - BAC3	1.97	20.98	8.3
S19 - BAC4	2.03	13.80	8.8
S19 - BAC5	1.97	16.33	8.7
**Control**			
S51-T1	1.87	0.71	ND
S51-T2	2.12	6.16	9.6
S51-T3	2.14	8.50	9.5
S51-T4	2.11	2.85	8.9
S51-T5	2.11	7.64	9.7
S52-T1	2.11	7.82	9.2
S52-T2	2.14	10.40	9.5
S52-T3	2.13	10.32	9.5
S52-T4	2.1	9.14	9.3
S52-T5	2.12	11.71	9.6
S53-T1	2.12	9.87	9.4
S53-T2	2.13	10.46	9.6
S53-T3	2.12	10.68	9.6
S53-T4	2.12	8.22	9.5
S53-T5	2.12	12.02	9.3
S54-T1	2.1	7.80	9.5
S54-T2	2.13	4.32	9.5
S54-T3	2.11	5.41	9.7
S54-T4	2.09	6.04	9.7
S54-T5	2.11	5.12	9.7
S56-T1	2.09	14.35	9.9
S56-T2	2.12	13.00	9.3
S56-T3	2.12	14.85	9
S56-T4	2.11	12.50	9.5
S56-T5	2.11	14.05	9.3

NA-Not applicable, sample failed
ND-not done
