# Supplementary material for: Microarray characterization of gene expression changes in blood during acute ethanol exposure
Source: BMC Med Genomics. 2013 Jul 25;6:26. doi: 10.1186/1755-8794-6-26 (PMC3750403; doi:10.1186/1755-8794-6-26)
Supplement: Additional file 2 — Primers used in study. [file 1755-8794-6-26-S2.pdf]

Additional File 2. Primers used in study

Refseq Accession ID	Affymetrix Probeset ID	Gene	Sense Primer	Anti-sense Primer	Intron spanning	Amplicon Size(bp)	*Position 5'	Position 3'	Transcript Length(bp)	Affymetrix Target	Cycling temperature	Primer concentration S/A uM
**Normalizers**												
NM_002205	201389_at	ITGA5	TCAGACATTGGCACCTAATC	TTCCTGGCTTCTCCTAAATC	NO	104	3879	3982	4248	3725-4193	54	300/300
NM_002661	204613_at	PLCG2	GCAGATGAATCACGCATTG	GAGCACCGAGAACCTTG	YES	149	3215	3363	4263	3666-4214	54	300/200
NM_005697	218143_s_at	SCAMP2	GGACAGCGGTTGGATTG	GAGAGCACGGCACAGAG	YES	108	803	910	1292	818-1150	54	300/200
**Genes of Interest**												
NM_015199	226025_at	ANKRD28	CATAAATTAAGCATCACTAAAGTCTC	CCGAAATATCAGCCTTCTCTC	NO	199	4379	4577	6339	4387-4920	54	250/300
NM_014210	204774_at	EVI2A	ACAAGTAGGCAAGCGTCAG	TGTGAGCACTCCAGTAGATTG	NO	139	866	1004	1572	994-1486	57	250/250
NM_004117	224840_at	FKBP5	TTTAAAAGCCCCAGTCTCACC	CACACACCCTTCCCCTCCC	NO	256	3257	3522	3772	3262-3678	57	150/200
NM_014020	220532_s_at	LR8	GCAGAAGGAGGAGTGTAGAG	ACAGGACAATGGCAGTGG	YES	251	849	1099	1330	739-1271	57	250/250
NM_014372	208924_at	RNF11	CCTCTTGTCTTGAACCATAG	ATTCTGCGTTTGTGTGC	NO	196	2197	2374	3139	2143-2656	53	200/200
NM_021259	221882_s_at	TMEM8	CCCTGCCTCTTTGCCTTC	TAGTAGTAGTTGTCGCTAGTCATC	YES	179	2067	2245	2530	1953-2508	57	250/250
NM_001785	205627_at	CDA	GTGACAGCCAGAGAATGCC	CGGTGTTCTAAGTCCCAAGG	NO	192	617	808	957	372-882	57	250/250
NM_014214	203126_at	IMPA2	GATTAACTATGGGCGGGATG	CCAGCCACTCACTGTAGC	NO	182	1051	1232	1500	950-1336	58	300/250

*Position numbers refer to the refseq transcript.
S/A sense/antisense
