# Supplementary material for: Microarray characterization of gene expression changes in blood during acute ethanol exposure
Source: BMC Med Genomics. 2013 Jul 25;6:26. doi: 10.1186/1755-8794-6-26 (PMC3750403; doi:10.1186/1755-8794-6-26)
Supplement: Additional file 3 — K means expression cluster members. [file 1755-8794-6-26-S3.pdf]

Additional File 3. K means expression cluster members.

1		2		3		4		5		6		7		No Cluster	
Probe Set ID	Gene Symbol	Probe Set ID	Gene Symbol	Probe Set ID	Gene Symbol	Probe Set ID	Gene Symbol	Probe Set ID	Gene Symbol	Probe Set ID	Gene Symbol	Probe Set ID	Gene Symbol	Probe Set ID	Gene Symbol
1557987_at	---	226392_at	---	226399_at	---	1555303_at	---	1557852_at	---	232024_at	GIMAP2	204774_at	EVI2A	221541_at	CRISPLD2
1566959_at	---	226641_at	---	226444_at	---	235735_at	---	228854_at	---	213831_at	HLA-DQA1	224731_at	HMGB1	202887_s_at	DDIT4
222375_at	---	229629_at	---	203156_at	AKAP11	204265_s_at	GPSM3	242695_at	---	209480_at	HLA-DQB1	225519_at	PPP4R2	206302_s_at	NUDT4
235028_at	---	243495_s_at	---	226025_at	ANKRD28	34031_i_at	KRIT1	229164_s_at	ABTB1	212509_s_at	MXRA7	223809_at	RGS18	202917_s_at	S100A8
242874_at	---	221505_at	ANP32E	220940_at	ANKRD36B	207079_s_at	MED6	1555736_a_at	AGTRAP			218340_s_at	UBA6		
1558015_s_at	ACTR2	221230_s_at	ARID4B	212614_at	ARID5B	201502_s_at	NFKBIA	200078_s_at	ATP6V0B						
200612_s_at	AP2B1	203427_at	ASF1A	209903_s_at	ATR	220001_at	PADI4	225373_at	C10orf54						
211833_s_at	BAX	208861_s_at	ATRX	203531_at	CUL5	200919_at	PHC2	234926_s_at	C20orf43						
223559_s_at	C9orf80	202265_at	BMI1	203302_at	DCK	205863_at	S100A12	205789_at	CD1D						
1556389_at	CNPY3	224740_at	C5orf43	205000_at	DDX3Y			205627_at	CDA						
225140_at	KLF3	200934_at	DEK	222850_s_at	DNAJB14			203973_s_at	CEBPD						
201551_s_at	LAMP1	209187_at	DR1	219279_at	DOCK10			205382_s_at	CFD						
230528_s_at	MGC2752	212149_at	EFR3A	225290_at	ETNK1			206380_s_at	CFP						
221771_s_at	MPHOSPH8	227462_at	ERAP2	227239_at	FAM126A			202121_s_at	CHMP2A						
207815_at	PF4V1	217941_s_at	ERBB2IP	201889_at	FAM3C			222934_s_at	CLEC4E						
226310_at	RICTOR	217234_s_at	EZR	226811_at	FAM46C			217870_s_at	CMPK1						
202131_s_at	RIOK3	229460_at	FAM126B	203115_at	FECH			201360_at	CST3						
226825_s_at	TMEM165	233898_s_at	FGFR1OP2	225153_at	GFM1			204971_at	CSTA						
235798_at	TMEM170B	218313_s_at	GALNT7	220577_at	GVIN1			203028_s_at	CYBA						
218345_at	TMEM176A	221510_s_at	GLS	202983_at	HLTF			222429_at	DBNL						
240383_at	UBE2D3	224862_at	GNAQ	205884_at	ITGA4			226064_s_at	DGAT2						
208882_s_at	UBR5	221763_at	JMJD1C	1553530_a_at	ITGB1			219452_at	DPEP2						
204619_s_at	VCAN	226675_s_at	MALAT1	226635_at	LOC401504			201041_s_at	DUSP1						
		221760_at	MAN1A1	228841_at	LYRM7			217992_s_at	EFHD2						
		218499_at	MST4	201151_s_at	MBNL1			203274_at	F8A1 /// F8A2 /// F8A3						
		212530_at	NEK7	226039_at	MGAT4A			218831_s_at	FCGRT						
		227354_at	PAG1	224830_at	NUDT21			224840_at	FKBP5						
		244008_at	PARP8	221751_at	PANK3			205119_s_at	FPR1						
		226119_at	PCMTD1	202741_at	PRKACB			200645_at	GABARAP						
		222572_at	PDP1	218236_s_at	PRKD3			217755_at	HN1						
		213111_at	PIKFYVE	202677_at	RASA1			205403_at	IL1R2						
		240413_at	PYHIN1	222605_at	RCOR3			203126_at	IMPA2						
		212636_at	QKI	208924_at	RNF11			204169_at	IMPDH1						
		222717_at	SDPR	218738_s_at	RNF138			202531_at	IRF1						
		212887_at	SEC23A	203403_s_at	RNF6			226267_at	JDP2						
		1553177_at	SH2D1B	226682_at	RORA			218963_s_at	KRT23						
		225564_at	SPATA13	212179_at	SFRS18			217388_s_at	KYNU						
		230836_at	ST8SIA4	213649_at	SFRS7			228648_at	LRG1						
		212956_at	TBC1D9	236265_at	SP4			201412_at	LRP10						
		221428_s_at	TBL1XR1	226529_at	TMEM106B			1559502_s_at	LRRC25						
		224793_s_at	TGFBR1	217826_s_at	UBE2J1			236172_at	LTB4R						
		202606_s_at	TLK1	224691_at	UHMK1			205859_at	LY86						
		232591_s_at	TMEM30A	202413_s_at	USP1			213708_s_at	MLX						
		202778_s_at	ZMYM2	218396_at	VPS13C			203936_s_at	MMP9						
				227309_at	YOD1			204961_s_at	NCF1 /// NCF1B /// NCF1C						
				222731_at	ZDHHC2			223944_at	NLRP12						
				226015_at	ZNF12			218388_at	PGLS						
								41469_at	PI3						
								201400_at	PSMB3						
								211178_s_at	PSTPIP1						
								203175_at	RHOG						
								213566_at	RNASE6						
								224573_at	RNASEK						

				203379_at	RPS6KA1			
				200660_at	S100A11			
				203186_s_at	S100A4			
				217728_at	S100A6			
				203535_at	S100A9			
				1554624_a_at	SIRPB1			
				1559034_at	SIRPB2			
				201463_s_at	TALDO1			
				208700_s_at	TKT			
				223482_at	TMEM120A			
				221882_s_at	TMEM8A			
				207643_s_at	TNFRSF1A			
				202096_s_at	TSPO			
				208864_s_at	TXN			

Calcium-binding
